# Monitoring Benzene, Toluene, Ethylbenzene, and Xylene (BTEX) Levels in Mixed-Use Residential-Commercial Buildings in Shiraz, Iran: Assessing the Carcinogenicity and Non-Carcinogenicity Risk of Their Inhabitants

**DOI:** 10.3390/ijerph19020723

**Published:** 2022-01-10

**Authors:** Zahra Baberi, Abooalfazl Azhdarpoor, Mohammad Hoseini, Mohammadali Baghapour, Zahra Derakhshan, Stefanos Giannakis

**Affiliations:** 1 Department of Environmental Health Engineering, School of Health, Shiraz University of Medical Sciences, Shiraz 7153675541, Iran; zahrababeri3@gmail.com (Z.B.); mohhoseini@sums.ac.ir (M.H.); baghapour@sums.ac.ir (M.B.); derakhshz@sums.ac.ir (Z.D.); 2 Research Center for Health Sciences, Institute of Health, Shiraz University of Medical Sciences, Shiraz 7153675541, Iran; 3 Unidad docente Ingeniería Sanitaria, Departamento de Ingeniería Civil: Hidráulica, Energía y Medio Ambiente, Universidad Politécnica de Madrid, 28040 Madrid, Spain

**Keywords:** BTEX, indoor air, risk assessment, residential-commercial buildings

## Abstract

The aim of this study is to investigate the concentration of Benzene, Toluene, Ethylbenzene, and Xylene (BTEX) compounds in the indoor air of residential-commercial complexes and to compare it with other residential buildings (control) as well as to assess the carcinogenicity and non-carcinogenicity risk of these pollutants. BTEX concentration was investigated in the indoor air of 30 ground floor restaurants, 30 upper residential units of the complexes, 20 adjacent residential units (control), and their corridors. The mean BTEX concentration measured in the upper residential units was reported higher than in the control residential units, though they were not significantly different. The lifetime cancer risk (LTCR) value calculated for benzene in the upper residential units was lower than 10^−4^ and higher than 10^−6^ across all ages, indicating a carcinogenicity risk. Furthermore, the mean hazard quotient (HQ) for all compounds was obtained lower than 1, suggesting no concern about the non-carcinogenicity risk of these compounds in the studied region. Nevertheless, considering the sources of benzene production in the indoor air as well as the carcinogenicity of these pollutants and the risk they pose in human health, application towards the reduction of the sources and concentration of benzene in the indoor air are necessary.

## 1. Introduction

One of the most important factors affecting human health is air pollution. Indeed, air pollution has recently claimed the highest importance among the environmental risk factors affecting human health [1]. Given these concerns, there are serious indications for the role of indoor air pollution. Since people spend a short period of their time outside the house (<20%), exposure to the indoor air pollutants becomes increasingly relevant [2]. There is a direct relationship between the human health and the quality of the indoor air of the building in which they live [3]. Benzene, Toluene, Ethylbenzene, and Xylene (BTEX) compounds have often been used as an indicator of the air quality in indoor environments, and include benzene, toluene, ethylbenzene, and xylenes [4]. These compounds constitute a group of aromatic hydrocarbons with special environmental properties, which can cause chronic toxicity even at low concentrations [5]. Generally, BTEX compounds are utilized as an index for exposure to volatile organic compounds, as well as oil compounds. Exposure to BTEX compounds may occur through ingestion (consuming contaminated water with BTEX), inhaling polluted air, or absorption through the skin [6]. 

BTEX concentrations at various climatic conditions depend on different factors including the mixing ratio with their surrounding gases, water solubility and Henry’s law constant, frequency and intensity of precipitation, gas and water level interaction, content, and concentration of other species in the atmospheric air, as well as the origin of air masses [7]. BTEX compounds have been long classified as priority pollutants according to the American Environmental Protection Agency [8], and yet, decades later their importance is still relevant. Two important features of BTEX are their existence in the ambient air and their association with their atmospheric concentrations [9]. The second point is utilized in BTEX studies to determine the photochemical age and sources of these compounds. According to EPA, the concentration of pollutants in indoor air is significantly higher than the level outdoors. In open spaces, pollutants become diluted because of their dispersion across the air, but in indoor environments, due to the lack of suitable ventilation and high humidity, the concentration of pollutants grows dramatically [10]. Furthermore, BTEX compounds are encountered at high concentrations in many indoor and residential areas because of their high vapor pressure [11]. 

Exposure to these compounds may have considerable impact on human health. These compounds are associated with chronic asthma, cancer, as well as some neurological disorders and symptoms such as weakness, loss of appetite, fatigue, dizziness, nausea, and even burning sensation in the eyes, skin, mucous membranes, and respiratory system [12]. Benzene is the most toxic chemical in the BTEX family, and long-term exposure to this compound may increase the chance of leukemia and aplastic anemia [13]. The International Association of Research on Cancer (IARC) has classified benzene as ‘human carcinogen’ in the group 1 of carcinogens, and ethylbenzene in group IIB ‘possible human carcinogen’ [14]. Thus, studies focusing on monitoring, risk assessment and methods for reducing or eliminating the risks of indoor air pollutants, thereby maintaining public health safety, must be undertaken. 

Among the existing gaseous pollutants, benzene, toluene, ethylbenzene, and xylene (BTEX) constitute a group of hazardous pollutants which has been a hot topic of research worldwide [11,15,16]. In Spain, Esplugues et al. showed that the average concentrations of benzene, toluene, ethylbenzene, ortho-xylene, and meta- and para-xylene were 0.9, 3.6, 0.6, 0.6, and 1.0 μg/m^3^, respectively. They found that the indoor levels of all the compounds were approximately 2.5 times higher than those observed outdoors [17]. In Iran, Hazrati et al. showed that the concentrations of benzene were considerably higher than the recommended value of 5 μg/m^3^ established by the Iranian Environmental Protection Organization. Additionally, they found that, among the BTEX compounds, benzene (HQ = 0.51) and xylene (HQ = 0.47) had notable hazard quotients and were the main pollutants responsible for high hazard index in the monitored homes. Their results showed considerably high cancer risk for lifetime exposure to the indoor and outdoor benzene [18]. In Iran, Baghani et al. measured BTEXs at beauty salons. The obtained results showed that ethylbenzene had the highest concentrations in the salons among the BTEX. Only xylene had presented non-cancer risk, but benzene and ethylbenzene had remarkably higher cancer risk [4].

Furthermore, Hadei et al. found that wall coating, ventilation system, heating system, flat level, and distance from highways explained approx. 29, 60, 16, 60, and 59% of the BTEX concentrations, respectively. In addition, the health risk assessment showed that the carcinogenic risks of benzene and formaldehyde exceeded the threshold of 1 × 10^−4^ and represent a definite risk [19]. Mehralipour et al. showed that the average concentrations of benzene, toluene, ethyl benzene, o, m-Xylene, and p-Xylene were 6.45, 7.02, 10.07, 7.21 and 8.36 mg/m^3^, respectively. They stated that the mean cancer risk for benzene was estimated as 529 × 10^−5^ and the mean non-carcinogenic risks for toluene, ethylbenzene and o, m-Xylene, and p–Xylene (TEXs) were 17.57, 5.03, 24.03 and 27.88, respectively. Furthermore, they found that the cancer risk for benzene, and the non-carcinogenic risk for TEXs were much higher than in the recommended limits [20]. In Iran, Dehghani et al. showed that among the BTEX, benzene has the highest concentration in gyms. The mean of inhalation lifetime cancer risk (LTCR) for benzene in indoor air of gyms in their study was calculated 4.1 (10^−7^), which is lower than the standard limit set by the US Environmental Protection Agency and the World Health Organization. Accordingly, benzene imposes no risk for athletes in gyms investigated in their study [21]. Heydari et al. showed that the concentration of BTEX compounds in the indoor air of tobacco cafes in this city is significantly high, so that it can pose a serious risk to the health of employees and customers. They found that the cafes located in the basement, due to poor ventilation or the complete lack of it, accumulate large amounts of these pollutants and further endanger the health of customers. In addition, the risk assessment showed that the carcinogenic and non-carcinogenic risk levels of the air inside the tobacco cafes exceeded the safe limits recommended by the EPA [22].

In the ground floors of some residential areas, there are commercial units such as restaurants and canteens, which create numerous problems for the residents such as different volatile contaminants and odors. As a result of their importance, the aim of this study is to investigate the concentration of BTEX compounds in the indoor air of residential-commercial complexes and to compare it with other residential buildings (control). Accordingly, sampling campaigns in winter and summer across different areas of Shiraz city were performed. To investigate the public health effects, the carcinogenicity and non-carcinogenicity risk assessment of these pollutants on the residents of the residential-commercial complexes has been performed, and the potential risks are analyzed, identifying the existing use and construction-specific underlying correlations.

## 2. Materials and Methods

### 2.1. Sampling

**Figure 1:**
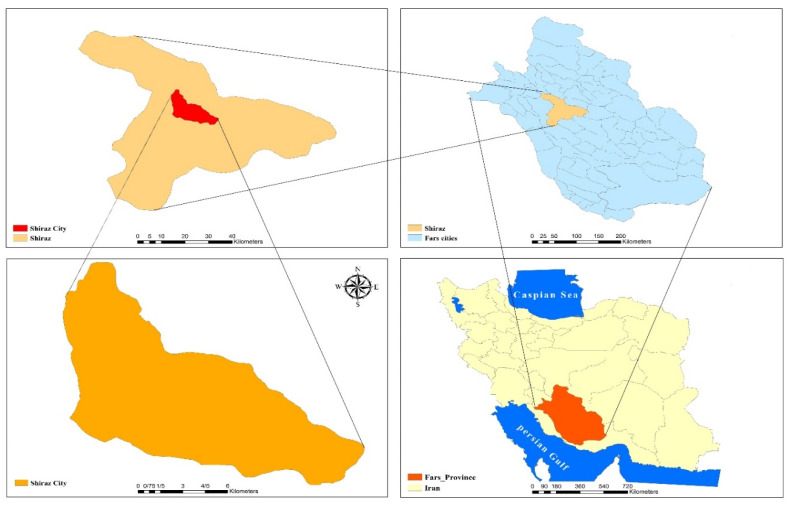
The general view of the location of Shiraz city and the studied region.

The general view of the location of Shiraz city, situated in the Fars province in the southwest Iran has been displayed in Figure 1. According to the satellite images, its coordinates are 29°37′30″ N, 52°32′0″ E. The subject area is with warm climate and an annual average of 18 °C in which the highest and lowest temperatures are 43 °C and −0.4 °C, respectively. The average indoor air temperature of the sampling sites was 25 °C. This study was performed in winter 2020 and summer 2020. BTEX concentration was examined in the indoor air of:

1. Upper residential units in residential-commercial complexes,
2. Fast food and restaurant units on the ground floor of the residential-commercial complexes, and
3. Residential units close to the commercial units (control) based on NIOSH standard.

In this study, a total of 90 specimens were randomly taken in the city. BTEX concentration was investigated in the indoor air of 30 ground floor restaurants, 30 residential units (upper floors), and 20 adjacent residential units (control). In addition, 10 samples were taken from the corridors of residential as well as residential-commercial complexes. For the sampling, micro-flow sampling pumps with flow rate 0.5 L/min and charcoal absorbent were employed. The sampling was performed for 30 min taking a 15-L sample from the living area of the units, at 150 cm height and during working hours. Extraction and analysis on them were performed for 72 h [18].

In addition to ambient measurements, other parameters such as age of the house, heating system, cooking devices, number of cooking sessions per week, being double-walled (or not), smoking, and other issues that could be associated with concentration of BTEX compounds were obtained through a questionnaire distributed among the residents. The pollution sources in this study are divided into two categories, which are: (a) External sources: e.g., motor vehicles and (b) internal sources: heating system (stove, heating radiator, stove-split, heating radiator-split), smoking (hookah, cigarette, other), pesticides, kitchen plan and the unit’s painting.

### 2.2. Extraction and Analysis

BTEX compounds were extracted from charcoal tubes using 2 mL of carbon disulfide. Vials containing CS2 and charcoal were shaken slowly for 20 min. Next, the solvent was transferred to GC vials, whereby the BTEX compounds were measured using gas chromatography (Varian Co., Palo Alto, CA, USA) equipped with FID detector using a capillary column (TRB-1ms, 30 m) (Varian Co., Palo Alto, CA, USA). Next, 1 µL samples were injected into the capillary column. The temperature of the injector and detector was adjusted at 250 and 300 °C respectively. Moreover, the oven temperature was set at 40 °C for 10 min and then 10 °C/min up to 230 °C [18].

The calculated detection limit (LOD) and quantification limit (LOQ) for the studied BTEX using GC system were in the range of 0.3 and 0.6 μg/m^3^ and 0.1 and 0.18 μg/m^3^, respectively. The LOD and LOQ limits for each of the BTEX compounds are presented in Table 1.

### 2.3. Statistical Analysis

In this study, before the analysis, firstly, data distribution in terms of normality was investigated. Since none of the information groups had normal distribution, non-parametric methods were thus used for their analysis. Mann–Whitney U and Kruskal–Wallis tests were used to explore the relationship between each of the concentrations in the residential units of the mixed-use complexes and control residential units. In this study, the significance level was considered <0.05. All the above analyses were performed using SPSS 23.

### 2.4. Calculation of the Risk of Carcinogenicity and Non-Carcinogenicity

To assess the risk of BTEX on human health, the carcinogenicity and non-carcinogenicity risk of these pollutants was calculated. To assess the risk of carcinogenicity over time resulting from inhaling benzene and ethylbenzene, firstly their chronic daily intake was calculated using the following formula (Equation (1)):

CDI = (CA × IR × CF × ED × EF)/(AT × BW)(1)

where CA describes the concentration of target pollutants (µg/m^3^), CF and IR are the conversion factor (mg/µg) and inhalation rate, respectively. In addition, ED (year), EF (days/year), BW (kg) and AT (= ED × 365 for noncarcinogenic effects and 70 × 365 for carcinogenic effects) are exposure duration, exposure frequency, body weight and average lifetime, respectively.

After obtaining the CDI (mg/kg/day), the lifetime cancer risk (LTCR) of benzene and ethylbenzene was be calculated according to Equation (Equation (2)):

Cancer Risk (CR) = CDI × CSF(2)

In this equation, CSF (mg/kg/day) represents the cancer slope factor (through inhalation). According to WHO guidelines, if the LTCR value is larger than 10^−5^, there is risk of carcinogenicity; also, according to the US-EPA guidelines, if LTCR is larger than 10^−4^, there is risk of carcinogenicity, and if it is within 10^−4^–10^−6^, there is probability of cancer risk.

Furthermore, to assess the risk of non-carcinogenicity over time, the hazard quotient (HQ) resulting from exposure to BTEX pollutant was calculated according to (Equation (3)):

HQ = CDI/RFC(3)

## 3. Results and Discussion

### 3.1. BTEX Concentration in the Indoor Air

**Figure 2:**
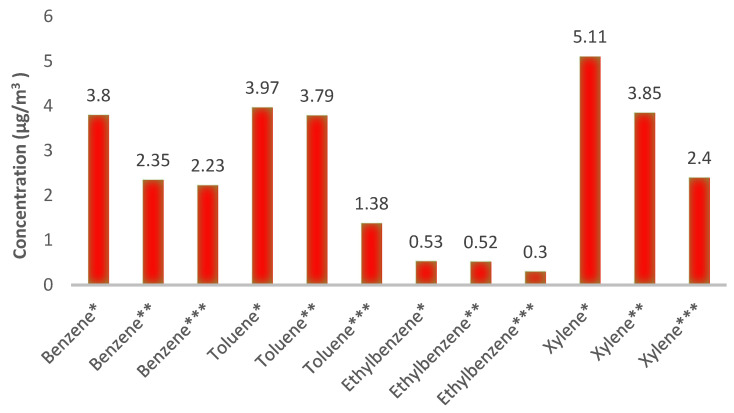
The mean concentration of BTEX compounds in the ground floor restaurant units (*), upper residential units of the residential-commercial complexes (**), and control residential units (***).

Figure 2 displays the values of BTEX concentration in the indoor air of residential and commercial complexes. The results indicate higher concentration of BTEX compounds in the indoor air of fast food and restaurant units in comparison to the indoor air of upper residential units. Furthermore, the concentration of BTEX compounds was higher in the indoor air of upper residential units compared to the control residential units. Table 2 summarizes the concentration distribution of the BTEX compounds in the indoor air of fast food and restaurant units, upper residential units of residential-commercial complexes, residential units (control) and corridor (corridor in residential-commercial complexes, and corridor in residential complexes).

Table 3 presents the results of Mann–Whitney and Kruskal–Wallis’ tests for the compared groups. The results indicate that there has been a significant difference between the (ground-floor) restaurants and its upper residential unit groups in terms of benzene, meta-xylene, and para-xylene concentration, as well as the sum of BTEX (*p*-value < 0.05). Nevertheless, the results of the test did not show significant differences for the upper residential units and control residential units, hence higher floors may present higher safety. It was also observed that among the studied pollutants in the corridor of the residential and residential-commercial complexes, there was a significant difference only in the concentration of meta-xylene and para-xylene. In addition, the results of Kruskal–Wallis’ test regarding the comparison of pollutant concentrations in winter (2020) and summer (2020) showed that there was a significant difference between the two seasons in terms of the concentration of benzene, ethylbenzene, meta-xylene, para-xylene, and ortho-xylene, which can be due to the use of heating systems during the winter.

The largest mean concentration of BTEX pollutant was observed in the restaurant units on the ground floor of the residential-commercial complexes, measured at 13.40 µg/m^3^, while for its upper residential unit was 10.51 µg/m^3^. The mean concentration of benzene throughout the study period across the upper residential units (2.35 µg/m^3^) was lower than that of the restaurant units and slightly higher than that of the control residential units (2.23 µg/m^3^). The significance of the Mann–Whitney U test results, when comparing the restaurant and the upper residential units, indicate the impact of distance from the sources of pollution emission on the concentration of the studied pollutants [19].

In the study by Baghani et al., after examining the indoor air of 50 beauty salons in Ardabil (Iran), among the BTEX compounds, ethyl benzene had the largest concentration (62.38 ± 32.37) in these establishments [4]. This concentration difference compared to our study can be due to the type of materials and solutions utilized in these salons, as well as the low air ventilation rate. However, in the study by Dehghani et al., among the BTEX compounds benzene claimed the largest concentration in the bodybuilding and fitness salons [21]. The mean BTEX concentration obtained in the present study was lower than the mean concentration of the study by Hadei et al. (53.2, 21.5, 14.4, and 21.1 µg/m^3^ for benzene, toluene, ethylbenzene, and xylene respectively) in the indoor air of residential buildings in Tehran, Iran. In their study, they noted the role of external environmental pollutant parameters, including the ventilation system, heating system, distance from the highway, as well as internal parameters such as the oil color of the walls, as well as parquet covering the units’ floor [20]. In our study, we collected several relevant parameters; Table 4 reports the descriptive statistics of several the questionnaire items (the number and percentage of each of the items), as well as the relationship between BTEX pollutants and the questionnaire items (construction parameters) in the control residential and residential-commercial units. Although smoking presented the highest contribution, no factor was considered dominant.

**Figure 3:**
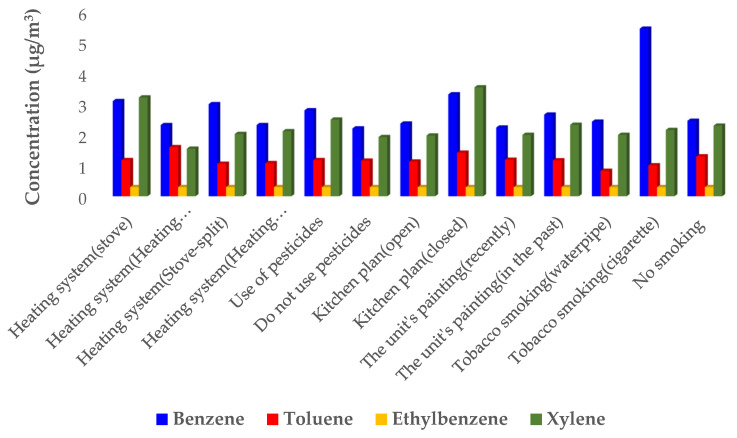
The relationship between the concentration of BTEX pollutant (µg/m^3^) and construction parameters in the upper residential units of the residential-commercial complexes.

**Figure 4:**
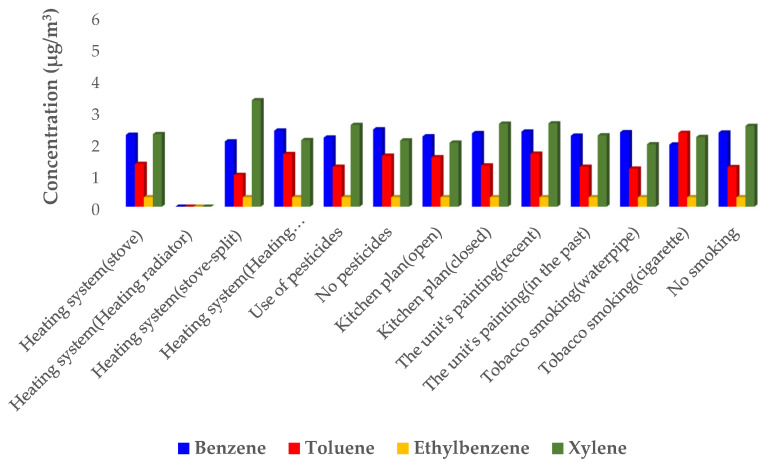
The relationship between the concentration of BTEX compounds (µg/m^3^) and construction parameters in the control residential units.

Figure 3 and Figure 4 reveal the relationship with the concentration of BTEX compounds and several construction parameters (questionnaire items). The mean benzene concentration (3.1 µg/m^3^) in the residential units of the residential-commercial complexes which used a stove was higher than those employing a heating radiator. Moreover, the mean benzene concentration was higher in the upper residential units in which the residents were smokers, as compared to other units. Similar results have been reported by previous studies in the production of benzene with smoking [20,22].

The mean concentration of BTEX in the residential units whose kitchen plan was closed was higher compared to the residential units of the residential complexes with an open kitchen design. Furthermore, the mean concentration of BTEX was higher in the residential units which used pesticides (6.78 µg/m^3^) as compared to the units that did not use pesticides (5.6 µg/m^3^). 

In the study by Esplugues et al. in Valencia (Spain), the level of all BTEX compounds was almost 2.5 times in the indoor air as large as the level in the open-air. The higher BTEX concentration in the indoor space depends on the buildings painting and lack of suitable ventilation. The higher concentration of BTEX in the open space is contingent upon location of the place of residence in the city center, residence in a region with higher traffic, and the season of the year [17]. Nevertheless, despite the differences between these parameters, no significant difference was observed in our study.

To determine the extent of the effect of emissions produced by motor vehicles (traffic), the benzene-to-toluene (B/T) ratio is used. Higher B/T is presented predominantly as a side-effect of traffic emissions [21], which is usually 0.3–0.8 [11]. The B/T = 0.62 in the present study indicated a considerable share of sources of emission by motor vehicles for BTEX. Thus, BTEX pollutants in the lower residential units can be affected by the external air pollution because of the proximity to streets, as well as the low air exchange rate and natural ventilation; the low wind speed close to the ground level plays a significant role in diluting the indoor air pollution of the building, as well as the pollutants emitted from the ground floor restaurant units.

### 3.2. Assessing the Health Effects of BTEX Presence

Table 5 presents the assessment of the carcinogenicity and non-carcinogenicity risk of BTEX pollutant and its comparison with other studies. According to Table 5, it is observed that the LTCR value calculated for benzene in the upper residential units and control has been 10^−4^–10^−6^ for all ages, which, according to the EPA instructions, can contribute to higher probability of cancer. Concerning the noncarcinogenic effects, the HQ value calculated for the residential units of the residential complexes and residential-commercial complexes was obtained lower than 1. In the study by Hazrati et al. in 2015, the LTCR value for benzene was calculated 125 × 10^−6^, suggesting its carcinogenicity risk [11].

In the study by Mehralipour et al., the mean estimated lifetime carcinogenic risk (LTCR) of benzene was 529 × 10^−5^, suggesting the carcinogenicity of this pollutant in the hookah cafés of Hamedan, Iran [20]. The study by Dehghani et al. indicated that among BTEX compounds, benzene had the largest concentration in the bodybuilding salons. However, the LTCR for benzene in the indoor air of the bodybuilding salons was calculated 4.1 × 10^−7^, which was lower than the standard limit determined by the World Health Organization and Environmental Protection Agency [21]. Furthermore, the mean HQ for all compounds was obtained lower than 1, suggesting no concern about non-carcinogenicity risk of these compounds in our studied region. However, in the study by Hazrati et al., the HQ value in the hookah cafés for ethylbenzene and xylene was estimated above 1 [11].

### 3.3. Considerations and Generalization Limitations

The disadvantage of this study is the data source. For example, in the case of painting there were a small number of monitored houses, where improvement projects coincided with the sampling timeframe. The limited number of questions in the questionnaire qs another limitation. In this study, we measured the concentrations of indoor BTEX at 30 ground floor restaurants and 30 residential units in Shiraz, Iran. Since the measurement at each unit was conducted at a different day, it is not possible to separately examine the temporal and spatial variations of the concentrations. Finally, the information regarding the indoor activity is obtained from a questionnaire distributed to the residents, hence the human factor has to be considered in the interpretation of the responses. Therefore, the accuracy of the information may vary from resident to resident, which may result in underestimation of the correlation.

## 4. Conclusions

The present study monitored the concentration of BTEX in the indoor air of mixed-use residential-commercial complexes (10.51 µg/m^3^) and its comparison with other adjacent residential units (control) (6.3 µg/m^3^). The mean benzene concentration in the upper residential units of the residential-commercial complexes was 2.35 µg/m^3^, which was slightly higher than the level of the control residential units. The LTCR value for the benzene indicated that there is possible risk of carcinogenicity due to its presence, because of exposure to this pollutant in the indoor air of residential-commercial complexes, although this effect is not definitive. Furthermore, the mean HQ for all compounds was obtained lower than 1, suggesting no concern over the non-carcinogenicity risk of these compounds. Since people spend more than 80% of their time in enclosed spaces, reduction of concentration and contact with hazardous pollutants, such as benzene, is necessary to achieve a lower load of disease and thereby reducing the national healthcare costs. Thus, attention should be paid to the quality of indoor air of residential-commercial complexes and introduction of monitoring air quality in such complexes.

## Figures and Tables

**Table 1:** LOD and LOQ limits in this study for the BTEX compounds.

	Benzene (μg/m^3^)	Toluene (μg/m^3^)	Ethylbenzene (μg/m^3^)	Xylene (μg/m^3^)
LOQ	0.3	0.6	0.3	0.6
LOD	0.1	0.18	0.1	0.18

**Table 2:** The mean and standard deviation for the studied units.

Pollutant Concentration (µg/m^3^)	Mixed Residential-Commercial Complexes						Control Residential Complexes			Corridors			
	Restaurants and Canteens			Restaurant Upper Residential Unit						Residential-Commercial		Control Residential	
	No.	x̅	σ	No.	x̅	σ	No.	x̅	σ	x̅	σ	x̅	σ
**BTEX**	30	13.40	13.44	30	10.51	13.26	20	6.32	1.59	7.17	4.32	4.44	1.29
**Benzene**	30	3.80	2.07	30	2.35	1.05	20	2.23	0.58	1.47	0.73	1.33	0.51
**Toluene**	30	3.97	9.58	30	3.79	9.87	20	1.38	0.61	1.10	0.42	1.04	0.10
**E-benzene**	30	0.53	0.77	30	0.52	0.79	20	0.3	0	0.3	0	0.3	0
**(m+p)-xylene**	30	4.24	5.12	30	3.03	3.44	20	2.10	1.06	3.82	4.26	1.25	0.71
**O-xylene**	30	0.87	2.81	30	0.82	2.54	20	0.3	0	0.47	0.38	0.5	0.16

**Table 3:** The results of Mann–Whitney and Kruskal–Wallis tests for the compared groups.

Pollutant	Mann–Whitney Test for the Ground Floor Commercial Unit and Upper Residential Unit	Mann–Whitney Test for the Upper Residential Unit and Control Residential Unit	Mann–Whitney Test for the Corridors of the Residential-Commercial Complexes and Control Residential Units	Kruskal–Wallis Test for the Relevant Concentrations in Winter and Summer
**BTEX**	0.002	0.91	0.22	0.20
**Benzene**	0.002	0.13	0.22	<0.0001
**Toluene**	0.049	0.43	1.0	0.52
**E-benzene**	0.72	0.99	1.0	<0.0001
**(m+p)-xylene**	0.001	0.51	0.032	0.04
**O-xylene**	0.95	0.99	0.31	<0.0001

**Table 4:** Descriptive statistics of the questionnaire items.

Questionnaire Items	Upper Residential Units of the Residential-Commercial Complexes			Control Residential Units
	Percentage (%)	*p*-Value	Percentage (%)	*p*-Value
**Heating system**				
Stove	16	0.66	50	0.39
Heating radiator	16		0	
Stove-split	12		15	
Heating radiator-split	56		35	
**Smoking**				
Hookah	12	0.09	20	0.93
Cigarette	24		15	
Other smokes	0		0	
None	64		65	
**Use of pesticides**				
Yes	52	0.89	60	0.08
No	48		40	
**Kitchen plan**				
Open	84	0.30	40	0.50
Closed	16		60	
**The unit’s painting**				
Recent	32	0.31	35	0.64
Old	68		65	

**Table 5:** Comparing the results of calculations for the carcinogenicity and non-carcinogenicity risk assessment of BTEX compounds in the present study and other studies.

Country	Location	Site		LTCR	HQ			Reference
				Lifetime (70 Years)	0–6 Years	6–21 Years	21–81 Years	
Iran	Shiraz	Residential-commercial buildings	Benzene	7.60 × 10^−6^	0.04	0.01	0.009	Present study (upper residential)
			Toluene	-	0.0003	0.0001	8.98 × 10^−5^	
			E-benzene	2.37 × 10^−7^	0.0002	0.0001	6.16 × 10^−5^	
			Xylene	-	0.18	0.07	0.04	
Iran	Shiraz	Residential-commercial buildings	Benzene	7.21 × 10^−6^	0.03	0.01	0.009	Present study (control residential)
			Toluene	-	0.0001	5.37 × 10^−5^	3.27 × 10^−5^	
			E-benzene	1.37 × 10^−7^	0.0001	5.83 × 10^−5^	3.55 × 10^−5^	
			Xylene	-	0.11	0.04	0.03	
Iran	Ardabil	Residential buildings	Benzene	3.21 × 10^−2^	0.51			[18]
			Toluene	-	0.014			
			E-benzene	3.63 × 10^−2^	0.012			
			Xylene	-	0.47			
Iran	Hamadan	Waterpipe cafes	Benzene	-	0.0057			[20]
			Toluene	-	0.043			
			E-benzene	-	0.015			
			Xylene	-	0.0104			
Iran	Tehran	Residential buildings	Benzene	Greater than 10^−4^	Greater than 1			[19]
			Toluene	-	Greater than 1			
			E-benzene	-	Greater than 1			
			Xylene	-	Greater than 1			
